# The migration percentage measured on EOS® standing full-leg radiographs: equivalent and advantageous in ambulant children with cerebral palsy

**DOI:** 10.1186/s12891-019-2746-2

**Published:** 2019-08-07

**Authors:** Jef Neirynck, Renee Proost, Anja Van Campenhout

**Affiliations:** University Hospitals Leuven, Herestraat, 49 3000 Leuven, Belgium

## Abstract

**Background:**

During ambulatory follow-up of patients with cerebral palsy (CP) systematic radiographic screening is required firstly to evaluate hip migration and development in the prevention of hip dislocation and secondly to analyse lower limb alignment and leg length. The Migration Percentage (MP) is a radiographic measurement used to describe the extent of femoral head lateralisation on conventional supine pelvic radiographs. Our goal was to assess the comparability of the MP measured on low radiation dose EOS® standing full-leg radiographs with that of conventional supine pelvic radiographs.

**Methods:**

Patients presenting with CP were prospectively selected from our outpatient follow-up consultation at our institutions CP reference centre and underwent conventional supine pelvic and EOS® standing full-leg radiographs the same day for diagnostic and screening reasons.

**Results:**

Out of 28 prospectively selected patients we included 21 (42 hips), of which 10 were female, with a mean age of 9.25 years and GMFCS levels of I, II and III. Seven out of 28 patients were excluded due to insufficient quality of radiographic images. The absolute differences in MP measured on both conventional supine pelvic and EOS® standing full-leg radiographs ranged between − 8 and 6% with an absolute mean difference of 0% (SD ±3.5) and were not statistically significant (*p* = 0.99). A Bland-Altman plot showed acceptable agreement between both measurements without proportional bias.

**Conclusion:**

There is no statistical significant difference between the Migration Percentage measured on conventional supine pelvic radiographs and EOS® standing full-leg radiographs in ambulant patients. These images use lower radiation doses and contain more radiographic information.

**Trial registration:**

Approved by the Medical Research Ethics committee of the University Hospitals Leuven (MP001492).

## Background

Due to femoral anteversion and limited bony remodeling of valgus in the femoral neck combined with both spasticity and weakness, common problems in children with cerebral palsy (CP) include hip dysplasia, progressive hip migration and risk for hip dislocation. Hip dislocation is present in 15 to 30% of patients with an even higher incidence rate in case of spastic quadriplegia. Increasing dysplasia will lead to hip joint pain, gait dysfunction, postural dysfunction, scoliosis and perineal hygiene problems [1–3]. Systematic radiographic and clinical screening allow for planning of preventive surgery preserving both hip coverage and function [4, 5].

Reimers described the Migration Percentage (MP) in 1980 as a tool to measure the extent of femoral head lateralisation [6]. He found previous methods such as the Centre Edge angle to be more position dependent and non-linear. Although Reimers estimated a standard measuring error of ± 10%, several authors published high inter- and intraobserver reproducibility [7–9]. The MP combined with other factors such as age and Gross Motor Function Classification System (GMFCS) has been found to be a good predictor of progressive hip migration and dislocation [10]. The MP is therefore extensively used in hip screening programs in children with CP.

The EOS® 2D/3D biplanar imaging system (Eos Imaging®, France) produces simultaneous antero-posterior (AP) and lateral 2D images allowing for construction of a 3D image based upon statistical models. It has been validated for study of the different parameters around the pelvis [11–13]. Inter- and intraobserver reproducibility proved to be very high compared to conventional radiographs [7, 14]. Radiation dose-area product is significantly lower compared to conventional full-leg radiographs as reported by Dietrich et al. (92.1 ± 45.5 cGy*cm2 vs 170.9 ± 104.2 cGy*cm2) [11].

**Fig. 1:**
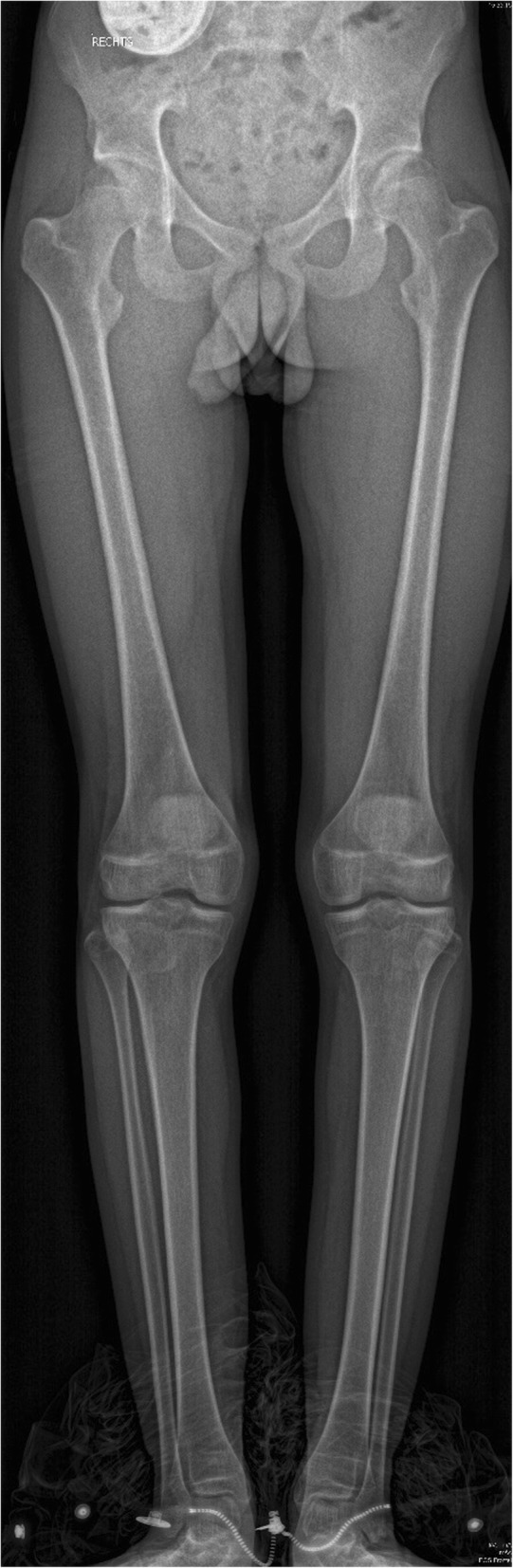
An example of an antero-posterior EOS® full-leg radiograph in a patient

During ambulatory follow-up at our department of paediatric orthopaedics (CP reference centre), a biplanar full-leg radiograph is often made in children with CP to evaluate lower limb alignment and leg length discrepancies (Fig. 1). We hypothesize obvious benefits from performing all measurements on just one radiograph to limit the number of radiographs, associated radiation dose, time, discomfort and financial cost to a minimum.

The goal of this study was to assess the comparability of the measurement of the MP on EOS® standing full-leg radiograph with that of conventional supine AP radiographs.

## Methods

### Patients

Twenty-eight Patients in need of radiographic full-leg evaluation, either for leg length discrepancy or lower limb alignment, as well as radiographic pelvic evaluation for hip screening were prospectively selected between September and October 2017 from our outpatient follow-up consultation at our institutions CP referral centre during which time the GMFCS level was verified. Inclusion criteria consisted of cerebral palsy, clinical need for both conventional supine AP pelvic radiograph and biplanar EOS® standing full-leg radiograph during the same day for diagnostic and screening reasons, between 2 and 18 years of age and correct radiographic measurements. Previous surgical intervention was no basis for exclusion.

### Radiographic imaging

The conventional AP pelvic radiograph was performed as described by Reimers in supine position with the hips in neutral position [6]. This was ensured by holding the knees neutral with the patella facing upwards. In cases with excessive lumbar lordosis and anterior pelvic tilt the hips were slightly flexed to reduce the pelvic tilt. The standing biplanar full-leg EOS® radiographs were performed in a routine procedure by experienced technicians. Hip and knee flexion was assessed on lateral images to ensure equal extension in both limbs. In case of a known leg length discrepancy the shortest limb was augmented to achieve a nearly horizontal pelvic alignment. All measurements were performed using Impax Viewer® (Agfa HealthCare NV, Belgium).

### Measurements

**Fig. 2:**
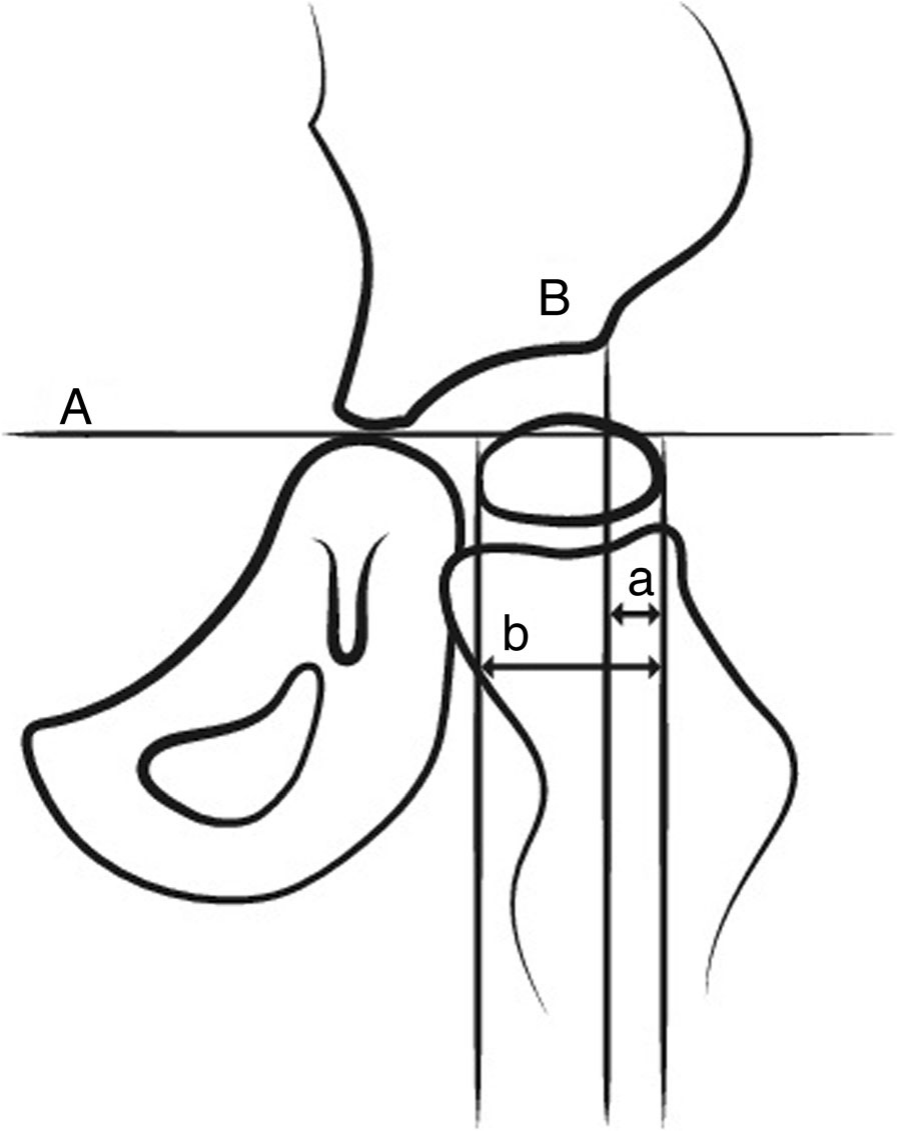
**a** represents Hilgenreiner’s line, a horizontal line connecting both tri-radiate cartilages. **b** represents Perkin’s line, a vertical line drawn at the lateral margin of the acetabulum perpendicular to Hilgenreiner’s line. The Migration Percentage is defined as the percentage of the femoral head situated lateral from Perkin’s line. It equates as ‘a/b’ with (**b**) representing the calcified femoral head at its widest point and (**a**) representing the part of (**b**) lateral from Perkin’s line

**Fig. 3:**
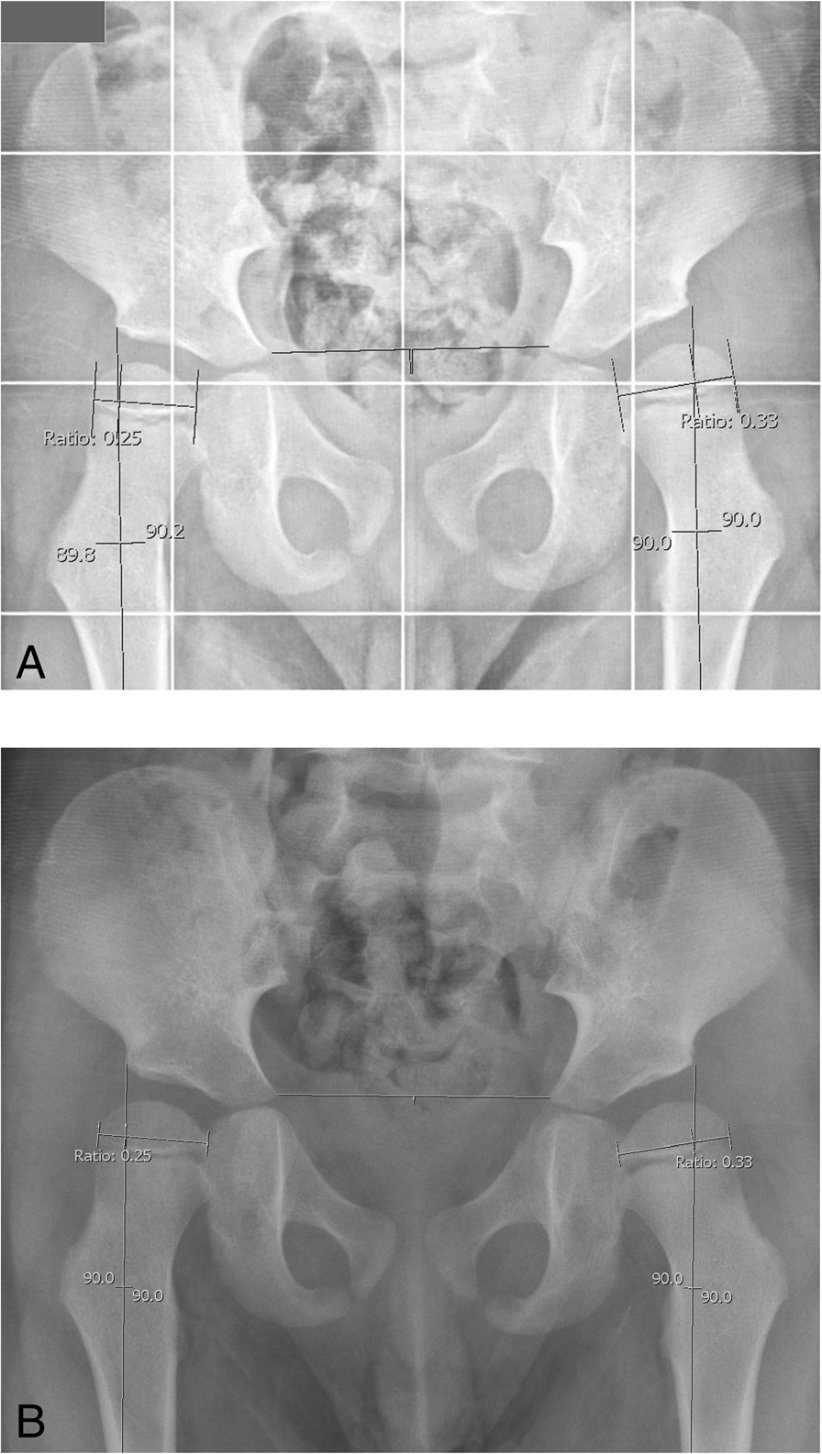
**a** and **b** Both images show a cut-out from radiographic images performed on a patient. Measurements were performed as displayed. **a** is cut from an EOS® full-leg AP radiograph. **b** is cut from a standard pelvic AP radiograph. The horizontal line represents Hilgenreiner’s line whereas the vertical line represents Perkin’s line. Reimers described the MP as the extent of the calcified femoral head at its widest point lateral from Perkin’s line divided by the total width of the calcified femoral head at its widest point reported as a percentage [6]. On these images the MP is 25% (0.25 × 100%) for the right hip and 33% (0.33 × 100%) for the left hip

The MP was the primary outcome measure. All measurements were performed by one orthopaedic resident (JN). However, to avoid bias all measurements were performed in order of patient inclusion, firstly measuring all conventional AP pelvic radiographs and reporting the results in a separate worksheet before measuring all full-leg EOS® radiographs. The MP is measured after drawing the Hilgenreiner’s line (a horizontal line connecting both tri-radiate cartilages) and Perkin’s line (a vertical line drawn at the lateral margin of the acetabulum perpendicular to Hilgenreiner’s line). It is defined as the percentage of the ossified femoral capital femoral epiphysis situated lateral of Perkin’s line (Figs. 2 and 3a and b).

### Statistical analysis

All statistical analysis was performed using SPSS (IBM corp®, version 25, US). Statistical significance was analysed using a non-parametric Wilcoxon Signed Rank Test. Normal distribution was assessed by Shapiro Wilks-test. Measurements were converted into a Bland-Altman plot to measure the agreement between both measurements.

### Ethics

Approval for this study was granted by the Medical Research Ethics committee of the University Hospitals Leuven (MP001492). No unnecessary radiographs were performed for research purposes. Only patients requiring both a pelvic examination and an assessment of leg length discrepancies were included with prior parental consent for both radiographs.

## Results

**Fig. 4:**
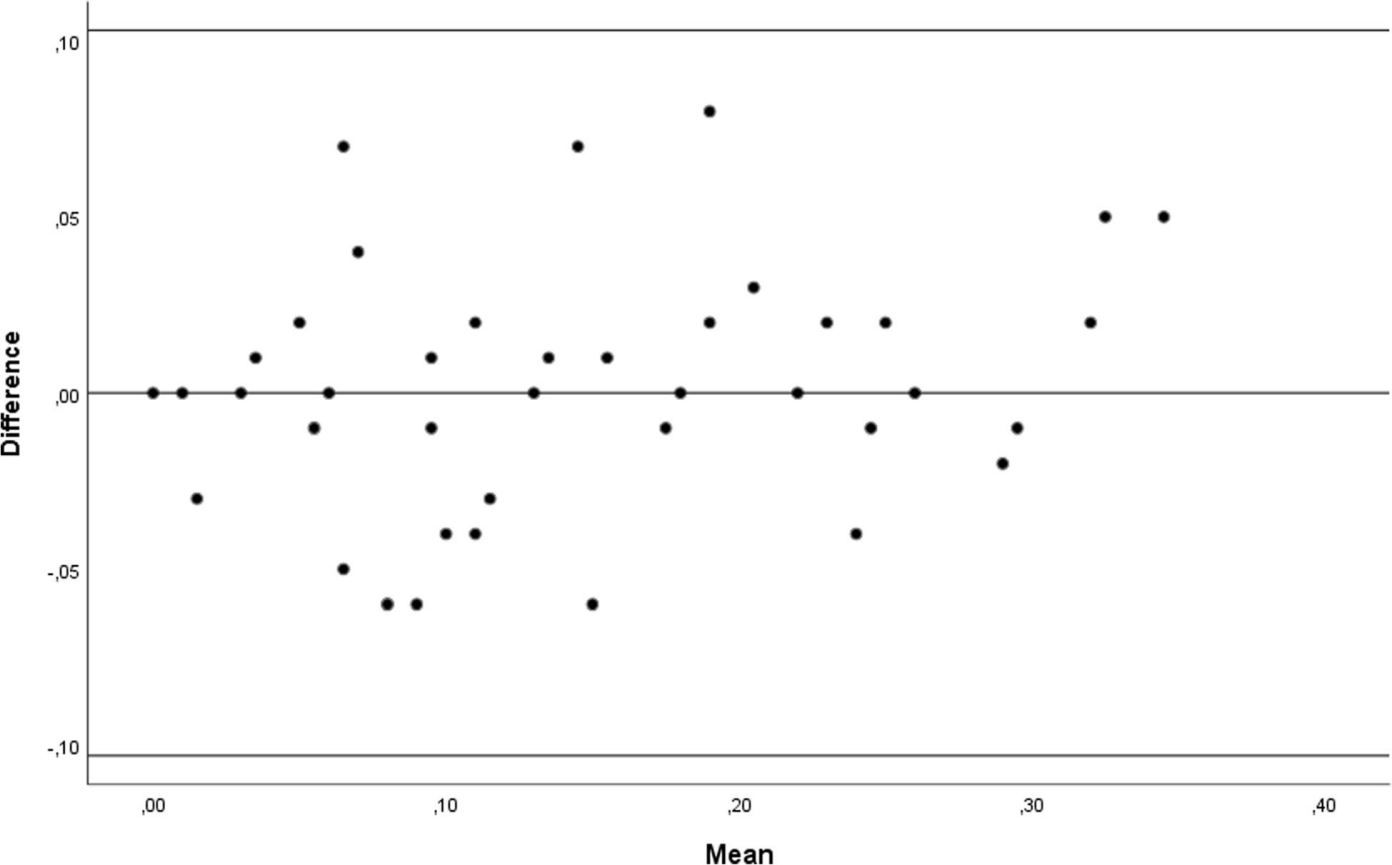
In this Bland-Altman plot all the dots are defined by the difference and the mean of both measurements from each hip. The overall mean difference is 0 (middle line). As all the dots are situated between the upper and lower ‘limit of agreement’ line (upper and lower line) we can accept good agreement between both measurements

Out of 28 prospectively selected patients 7 were excluded because of insufficient quality of radiographic images of which 4 due to covering of radiographic landmarks caused by the gonadal protection shield, 2 due to absent AP radiographs and 1 due to excessive movement whilst making the radiograph. Twenty-one patients, of which 10 were female (47.6%) were included with a mean age of 9.25 years. Eleven patients presented with a hemiplegia, 5 with diplegia and 5 with triplegia. Patients were classified according to the GMFCS as level I in five patients, level II in 13 patients and level III in three patients (Table 1). Mean MP was 14.4% (SD ±10%) in 42 hips assessed on EOS® standing full-leg radiographs compared to an identical 14.4% (SD ±9%) in 42 hips on supine pelvic radiographs. The absolute differences between measurements of the same hip ranged between − 8 and 6% with an absolute mean of 0% (SD ±3.5%). This was not statistically significant according to a Wilcoxon Signed Rank Test (*p* = 0.99). Post-hoc power analysis was impossible given the absolute mean difference and therefore effect size of 0%. To measure the agreement between both measurements we plotted all data into a Bland and Altman plot to visualize measurements of each hip according to the difference and mean between an upper and lower limit of agreement (overall mean difference ± 1.96 x SD) [15]. No measured differences were situated outside the limits of agreement without proportional bias (*p* = 0.098) between points above and underneath the mean difference line (Fig. 4).

**Table 1:** Stratification of the Migration Percentage measured in both standard and full-leg radiographs from all included patients according the different Gross Motor Function Classification System levels

GMFCS level	I	II	III	Overall
Number of patients	5	13	3	21
Mean age	10.05	10.25	8.63	9.91
Mean MP pelvic X-ray (%)	10	15	18	14
Mean MP full leg X-ray (%)	10	15	18	14
Mean absolute difference (%)	0	0	0	0

## Discussion

On many occasions a standing full-leg radiograph is performed to assess lower limb alignment or leg length discrepancies during ambulatory follow-up in children with CP. These patients also require regular radiographic hip screening. The comparability of measurement of the MP on EOS® standing full-leg radiographs with conventional supine pelvic radiographs was evaluated on a prospectively selected cohort of 21 patients (42 hips). As no statistical significant difference was found between the MP measured on both radiographs we assume the MP can be correctly measured on EOS® standing full-leg radiographs. The advantages offered by these images include reduced radiation doses and additional information regarding leg length and lower limb alignment. In this study the full-leg radiograph was taken using the EOS® imaging device, but the result of a reliable MP assessment equally applies for conventional standing full-leg radiographs. All the advantages above still apply except for reduced radiation doses.

Reimers estimated a standard measuring error of ± 10% due to the limited calcification of the femoral head in young children and difficulties in defining the lateral acetabular rim in larger children. Several authors already demonstrated a high inter- and intraobserver reproducibility and intraclass correlation coefficient when measuring the MP, these analyses were not performed as this was not the goal of this study [7–9, 16]. A recent study compared the classic MP with a modified MP using a vertical line at the lateral edge of the acetabular sourcil instead of the Perkin’s line. The classic method (as used in this study) proved to be more reliable [17].

Although the MP might be less position depended compared to the Centre-Edge angle, hip adduction, flexion and rotation might influence any measurement. It is essential that patient positioning and radiographic production must be performed in a standardized manner by experienced technician to achieve consistent and reproducible radiographs.

We were forced to exclude 7 patients for insufficient quality of radiographic images of which 4 due to covering of radiographic landmarks caused by the gonadal protection shield. Although this protection was applied with best intent additional radiographs and associated radiation were required. Therefore, we stress the importance of high-quality radiographic images. Inevitably we rely on the expertise and knowledge of the radiology technician for high quality images therefor good communication between the orthopaedic and Radiology department might limit the amount of unacceptable low-quality images.

We remark that since a standing full-leg radiograph requires the capacity to stand upright these images will be primarily indicated for ambulant patients classified as GMFCS level I, II & III. However these patients will exhibit less hip displacement [3]. Most children who require walking aids (GMFCS level III) are less ideal candidates for standing radiographs without standing support. Therefor children requiring the most intense hip screening (GMFCS level IV & V) can probably not be examined using a standing full-leg radiograph. Furthermore, very young children may not be able to stand alone in the EOS machine during the examination.

### Limitations

We acknowledge several limitations to this study. All measurements were performed by the same orthopaedic resident. Radiographic measurements are vulnerable to patient positioning and rotation. Flexion and internal rotation contractures influence the position of the proximal femoral head relative to the pelvis. Also, correcting internal femoral rotation is more difficult when the child is standing compared to a supine position. Finally, our sample size included 42 hips in ambulant children with CP with a relatively small hip migration.

## Conclusion

There is no statistical significant difference between the Migration Percentage measured on conventional supine pelvic radiographs and EOS® standing full-leg radiographs in ambulant patients. Therefore, the MP can be correctly measured on EOS® standing full-leg radiographs. These images use lower radiation doses and contain more radiographic information.

All procedures performed in studies involving human participants were in accordance with the ethical standards of the institutional and/or national research committee and with the 1964 Helsinki declaration and its later amendments or comparable ethical standards.

## Data Availability

The datasets generated and/or analyzed during the current study are not publicly available due the privacy of patients included but are available from the corresponding author on reasonable request.

## Abbreviations

CP: Cerebral Palsy
GMFCS: Gross Motor Function Classification System
MP: Migration Percentage
